# CO_2_-measuring dongle

**DOI:** 10.1016/j.ohx.2022.e00338

**Published:** 2022-07-14

**Authors:** Marc-Aurèle Boillat, Peter C. Hauser

**Affiliations:** University of Basel, Department of Chemistry, Klingelbergstrasse 80, 4056 Basel, Switzerland

**Keywords:** CO_2_-sensor, Air quality monitoring, Python, Forth

## Abstract

The compact pocketable CO_2_-measuring device is built on a small printed circuit board (PCB) with the dimensions of ca. 8.5 × 3 cm. It is plugged into the universal serial bus (USB) port of a personal computer (PC) which serves to provide power and for downloading the measurements. The measurements can be viewed on the computer display where they also can be plotted as a graph to recognize trends. As the level of CO_2_ rises indoors when people are present and the air is not exchanged adequately the device can be employed as a personal device for monitoring indoor air quality. This is of particular interest when airborne pathogens might be present such as during the COVID-19 pandemic. The device utilizes a novel compact CO_2_ sensor which has only become available commercially recently, and is based on the photoacoustic measuring principle. A graphical user interface on the PC programmed in Python allows easy interaction with the user. A MSP430FR2433 microcontroller on the board controls the sensor and establishes the communication to the software application on the computer. This was facilitated by the choice of Forth as the programming framework for the microcontroller.

## Graphical abstract

**Figure:**
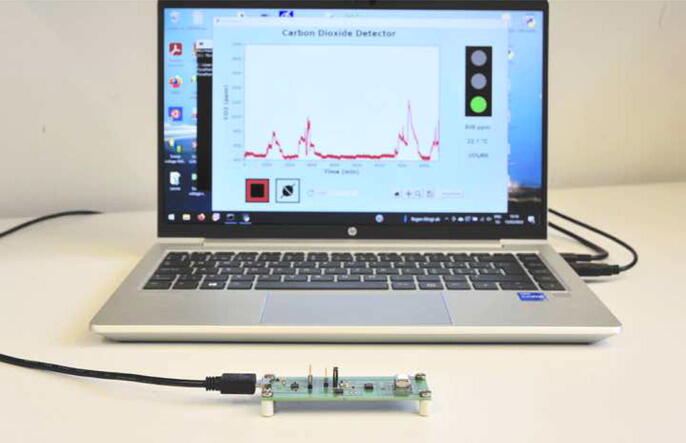

## 1 Hardware in context

Carbon dioxide (CO_2_) is released by humans through respiration and therefore when people are present in enclosed environments its concentration in air is found to increase from the ambient level of ca. 400 ppm. Relatively modest build-ups to 1000 ppm are already considered to lead to an impairment of human cognition, and higher levels can lead to drowsiness [1], [2], [3]. A lack of ventilation can also lead to an accumulation of airborne pathogens, and in light of the COVID-19 pandemic the World Health Organisation (WHO) therefore recommends rigorous standards of ventilation especially in classrooms [4]. CO_2_ levels are frequently measured as a general indicator for the effectiveness of ventilation [5], and indeed correlate with the risk of airborne infection [6], including the SARS-CoV-2 coronavirus [7]. The measurement of CO_2_ levels may serve as a monitor for the quality of ventilation.

Commercial devices for consumers to measure CO_2_ concentrations in air are readily available, often as part of instruments which also show other parameters such as temperature and humidity. However, these instruments are usually stationary, mostly need to be plugged into a mains power supply and feature a screen for direct display of the measurements. For this reason they are relatively large and not designed for mobile use. The device introduced herein is intended as a highly compact personal monitor which can be brought to indoor gatherings such as staff meetings, classes at schools or lectures at universities. As notebook computers are frequently carried by their users and brought to such settings anyway the device was designed as an attachment. This is a significant simplification as the notebook computer can then provide power to the measuring device and can serve for the read-out of the measurements, while the computer can still also be used for the normal tasks. The device therefore does not require its own power supply and display, and can therefore be kept much smaller and lighter than a stand alone unit. The computer screen also allows to show detailed information, i.e. a graph of a time series of readings. Furthermore, the measurements can be recorded on the computer for later reference and processing of the data. As the CO_2_-sensor device employed also incorporates temperature and humidity sensors this information is also available with the *CO_2_-dongle*. Sensirion, the manufacturer of the CO_2_-sensor used, has recently released a device, for CO_2_ measurements only, under the designation of *CO_2_-gadget*, which is also compact and powered from a USB supply. However, the two devices differ in the way the measurements are read out. For the *CO_2_-gadget* from Sensirion this is achieved with an app running on a smartphone which connects to the device via Bluetooth, while the *CO_2_-dongle* sends its results to a PC, which also provides the power. Besides its straight use as a CO_2_ monitoring device, the design reported herein may also be taken as a starting point if a CO_2_ sensor is to be integrated into a more complex monitoring or control system.

**Fig. 1:**
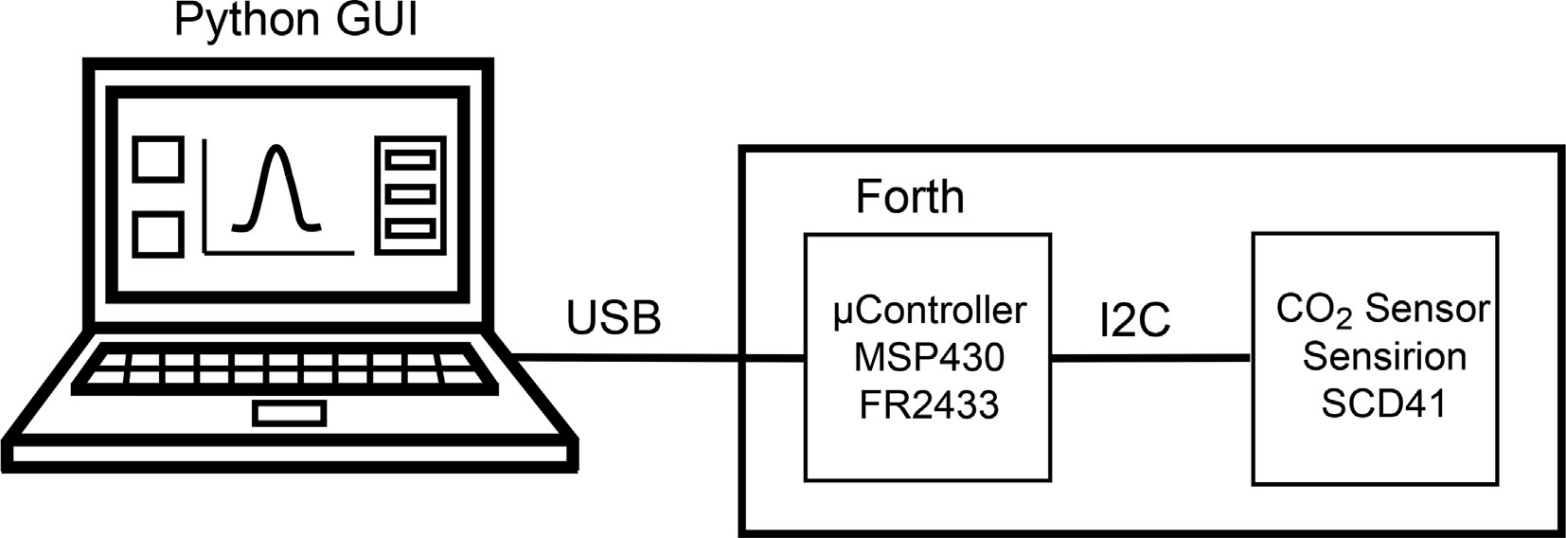
Block diagram of the set-up illustrating the communications between the Forth-microcontroller and the SCD41 sensor on the CO_2_-dongle via I2C and between the microcontroller and the PC via USB.

The *CO_2_-dongle* makes use of a microcontroller, which is connected to the PC via USB. An overview of the set-up is shown in Fig. 1. It is an excellent example of the tethered Forth-microcontroller approach for running electronic hardware from a PC as detailed in our earlier publication [8]. The microcontroller, in this case, enables the communication between the PC and the sensor, which has an I2C (Inter-Integrated Circuit) serial interface. Forth is an interpreter programming environment with a built in compiler, which in this case completely resides in firmware on the controller. Compiled subroutines (called *words* in Forth) and final programs are immediately executed when their name is passed to the microcontroller via its serial interface. This makes for an easy interaction with the attached computer, as it is not necessary to write a communication software for the microcontroller, which would be required if the more standard C/C++ language, as used on the Arduino platform, was employed. In the tethered approach, the complete Forth engine resides on a microcontroller with ports for interaction with experimental hardware. The PC can serve as a text based terminal for user interaction, or for routine use a graphical user interface (GUI) can be written. The Python software running on the PC then simply sends Forth words to the microcontroller via the USB port, and processes any data returned. Specific Forth words on the microcontroller developed for the CO_2_-sensor take care of its configuration and initiate measurements via the I2C serial interface. Forth allows for interactive testing of the subroutines while building a program, which greatly facilitated this task and minimizes the likelihood of introducing software bugs. The Python program on the computer essentially only has to handle the GUI and the sending and receiving of strings via the USB port. Writing the software for such a set-up is highly efficient as the task is divided up according to the different strengths of the two programming environments. Python comes with ready made libraries for creating the GUI and Forth excels in the low level hardware access. It may be argued that due to the similarity to standard Python used on the PC, it would be easier for someone skilled in this computer language to employ MicroPython on the microcontroller. However, Forth was developed in the 1970′s (incidentally at about the same time as C) when computer memory was limited and therefore it is ideally suited to run on current small microcontrollers. The complete Forth engine on the *CO_2_-dongle* requires 11 kBytes of memory, while MicroPython, even being only a stripped down version of Python, requires 256 kByte (see micropython.com). Therefore a much more powerful microcontroller would have been required to implement the *CO_2_-dongle* with MicroPython.

## 2 Hardware description

The CO_2_-measuring dongle is based on a single double-sided printed circuit board (PCB) with the dimensions of ca. 8.5 × 3 cm and after assembly has a weight of ca. 12 g.

• Communication to the computer is via USB.
• The USB port also provides the power to the dongle.
• The CO_2_-sensor includes built-in temperature and humidity transducers and these parameters are also read out.

**Fig. 2:**
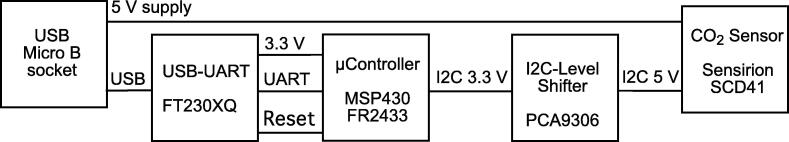
Block diagram of the circuitry on the CO_2_-dongle.

**Fig. 3:**
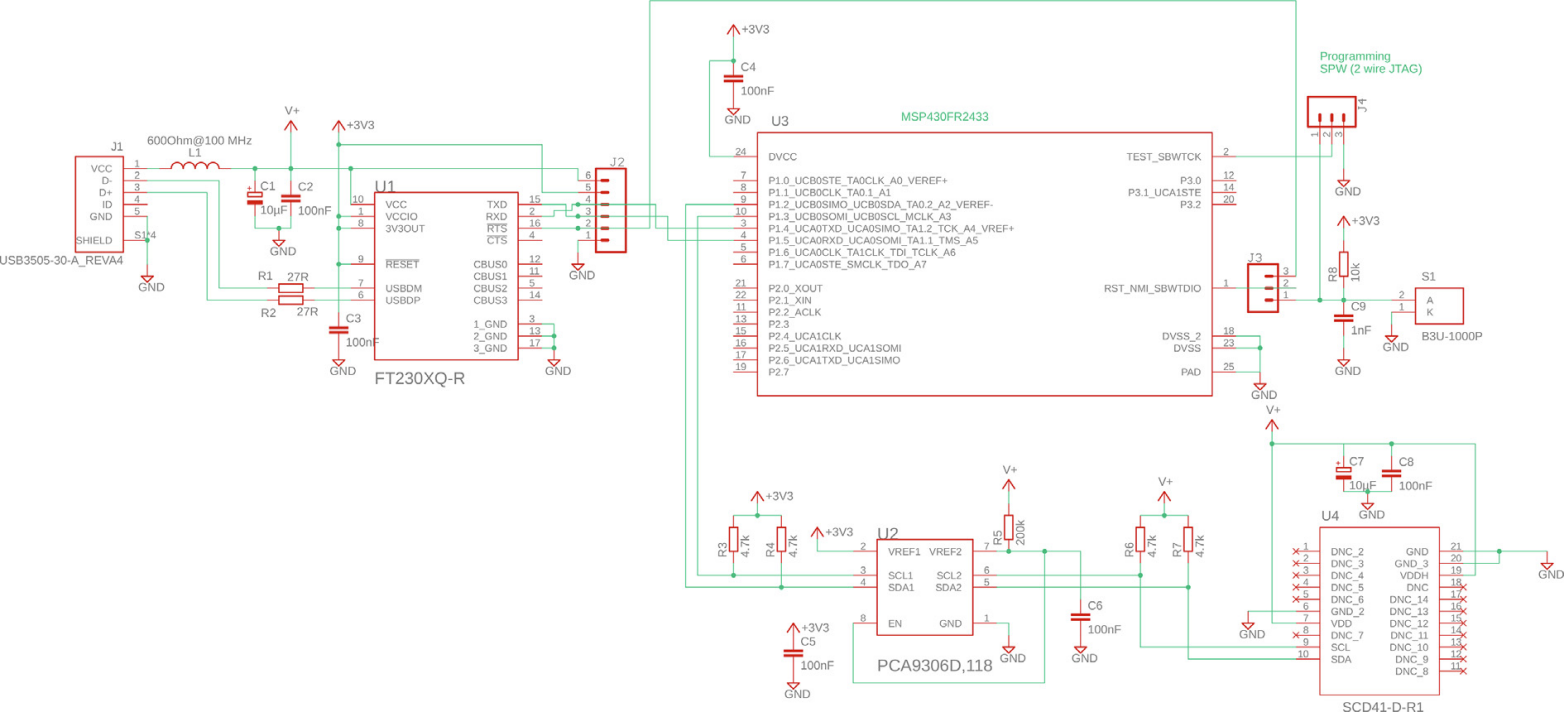
Circuit diagram created with EAGLE.

A detailed block diagram of the circuitry on the dongle is given in Fig. 2. The sensor itself is a novel device (SCD41) from Sensirion (https://sensirion.com) which achieves its unprecedented small dimensions for a CO_2_-sensor (10.1 × 10.1 × 6.5 mm) by utilization of the photoacoustic measuring principle. This sensor was therefore chosen in order to build a compact device as alternative sensors are based on infrared absorption measurements and require larger dimensions due to the necessary optical path length. For the photoacoustic sensor an intensity modulated infrared emitter inside its body excites acoustic waves on absorption of its energy, which are picked up by an acoustic transducer built into the device. Readers not familiar with photoacoustic gas sensing may wish to consult the recent reviews by Palzer [9] and by Yang et al. [10]. The sensor contains its own electronic circuitry, which includes an analog-to-digital convertor so that the measured signal is made available directly in digital format. Note also, that the SCD41 does not require calibration by the user, other than an exposure to atmospheric CO_2_ concentration at least once a week (see the manufacturer's data sheet for details). The microcontroller is a 16-bit MSP430 device (MSP430FR2433) from Texas Instruments with the Forth interpreter in firmware. The version of Forth employed is available as open source software under the designation of *Mecrisp* (https://mecrisp.sourceforge.net). Besides the sensor and the microcontroller a few other electronic components are required. To enable communication with the host computer a USB-to-UART bridge in form of a dedicated integrated circuit, the FT230XQ from FTDI is used. The sensor is powered directly by the 5 V of the USB bus, but the microcontroller is not 5 V compatible and is powered by 3.3 V, which is conveniently provided as an auxiliary by the FT230XQ. A level shifter IC to enable the I2C communication between the microcontroller and the sensor completes the basic set-up. A Micro B USB socket and auxiliary headers to allow the uploading of the firmware into the microcontroller complete the design. The detailed circuit diagram is shown in Fig. 3 and can be downloaded from the repository in the original format of the layout program EAGLE.

## 3 Design files

### 3.1 Design files summary

**Table:** 

Design file name	File type	Open source license	Location of the file
PCB.sch	EAGLE schematic	CERN OHL v.2.0	https://doi.org/10.5281/zenodo.6302824
PCB.brd	EAGLE layout	CERN OHL v.2.0	https://doi.org/10.5281/zenodo.6302824
Forth-Words.txt	Mecrisp Forth program	CERN OHL v.2.0	https://doi.org/10.5281/zenodo.6302824
GUI.zip	Python PC program	CERN OHL v.2.0	https://doi.org/10.5281/zenodo.6302824

PCB.sch and PCB.brd are the design files created with the printed circuit board layout program EAGLE for the circuitry.

Forth-Words.txt is the Forth programming to be installed on the MPS430FR2433 microcontroller.

GUI.zip contains the Python GUI software to be installed on the PC.

## 4 Bill of materials

**Table:** 

Designator	Component	Number	Cost per unit - €	Total cost - €	Source of materials	Material type
U1	FTDI FT230XQ-R	1	1.87	1.87	Mouser.com	semiconductor
U2	NXP PCA9306D	1	0.84	0.84	Mouser.com	semiconductor
U3	TI MSP430FR2433IRGER	1	2.11	2.11	Mouser.com	semiconductor
U4	Sensirion SCD41-D-R1	1	45.66	45.66	Mouser.com	CO_2_-sensor
J1	GCT USB3505-30-A-KIT	1	2.35	2.35	Mouser.com	Micro B USB 2.0 socket
	Qualtek 3025033–1	1	2.97	2.97	Mouser.com	USB cable A - Micro B
J2	Amphenol 77311–801-06LF	1	0.46	0.46	Mouser.com	male header
J3, J4	Amphenol 77311–801-03LF	2	0.25	0.50	Mouser.com	male header
	Preci-dip 999–19-220–00	1	0.40	0.40	Mouser.com	jumper 2.54 mm
L1	Würth Elektronik 742,792,040	1	0.19	0.19	Mouser.com	ferrite bead
S1	Omron B3U-1000P	1	0.92	0.92	Mouser.com	push button switch
C1, C7	Kemet T491A106K020AT	2	0.59	1.18	Mouser.com	10 µF capacitor 1206
C2, C3, C4, C5, C6, C8	Yageo CC0805ZRY5V9BB104	6	0.11	0.66	Mouser.com	100 nF capacitor 0805
C9	Kemet C1206C102K5REC7210	1	0.20	0.20	Mouser.com	1 nF capacitor 1206
	PCB with stencil	1	17.40	17.40	beta-layout.com	printed circuit board

For programming the microcontroller a MSP-FET programmer (€ 133.83, Mouser.com) or a LaunchPad (e.g. MSP-EXP430FR2433, € 11.63, Mouser.com), both from Texas Instruments, is required.

## 5 Build instructions

### 5.1 Printed circuit board and assembly

**Fig. 4:**
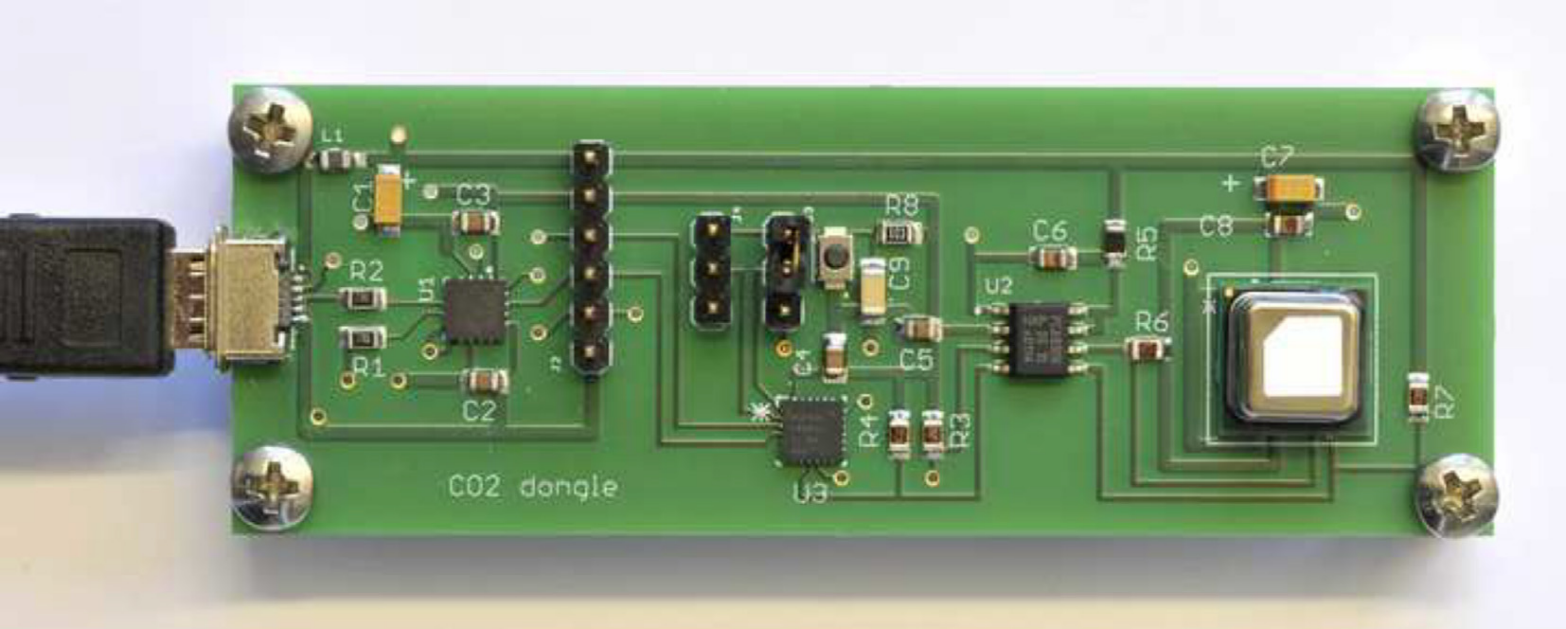
Photograph of the finished dongle showing from left to right: the USB connector, USB-UART-bridge IC, microcontroller, I2C level shifter IC and the sensor with the CO_2_-permeable membrane on top.

The double-sided printed circuit board was designed with the CAD program EAGLE (from https://www.autodesk.com), for which a free version is available for educational users. The PCB was ordered together with a stencil from Beta Layout (https://de.beta-layout.com), by submitting the EAGLE CAD file. Gerber files can be created from native EAGLE files if required. The population of the board with the surface mount devices has to be done by reflow soldering, following application of solder paste with the stencil and a small squeeqee. The components were placed with small tweezers under a magnifying glass, and soldered in a reflow oven, but the pin headers were soldered in last with a regular soldering iron. The finished board is shown in Fig. 4. If the PCB is placed into a case for robustness, generous ventilation holes will be required for the sensor (as a guiding figure, the *CO_2_-gadget* from Sensirion has a slot with an opening of ca. 30 mm^2^ on top of the sensor).

### 5.2 Programming of the FT230X USB-UART bridge

The FT230X is set-up correctly for communication between the PC and the microcontroller by default. However, on establishing the communication with the host PC it also informs the PC how much maximum current it should make available on the USB port used, which is then the limit allowed by the PC as long as the connection is established. A notebook PC is capable of delivering up to 500 mA, but the 90 mA requested by the FT230X by default is not sufficient for the peak current consumption by the sensor during the brief measurement periods when the IR emitter is turned on (according to the datasheet a maximum of 137 mA when operated from 5 V). Therefore this setting must be adjusted accordingly by changing the corresponding parameter stored in a non-volatile register on the FT230X. This is carried out with a Windows only program (FT_PROG) available for download from the manufacturer of the device (https://ftdichip.com). The instructions for use of this utility program can be found in the application note AN–124, which is also available from the website of the supplier. For this adjustment the dongle is simply connected to the USB port of the PC.

### 5.3 Programming of the microcontroller

First the Forth interpreter must loaded onto the MSP430FR2433 with the help of a hardware programmer for the MSP430 microcontroller family. For this purpose the manufacturer of the microcontroller (Texas Instruments) produces a device programmer with the designation MSP-FET, which can obtained from the major distributors of electronic components such as Mouser or Digi-Key. Alternatively a so-called Launchpad is used. These are evaluation boards from Texas Instruments for some of the MSP430 microcontrollers and contain a programming section. A Launchpad for the MSP430FR2433 is available, but any other MSP430 Launchpad should also work.

Download the mecrisp-2.0.7.tar.gz package from the Sourceforge repository (https://sourceforge.net/projects/mecrisp/files/). After expansion of the compressed package locate the forth-mecrisp-msp430fr2433.hex file.

The device programmer is used in conjunction with a PC and requires a software running on the PC. The most simple option is UniFlash, which is downloaded from Texas Instruments (https://www.ti.com) and available for PCs running Windows, macOS or Linux.

Connect the dongle to the computer in order to provide power to the microcontroller. Set the bridge on jumper 3 (J3) to connect pins 1 and 2. Connect the programming signals SBWTCK and SBWTDIO as well as GND from either the MSP-FET device programmer or the programming section of the LaunchPad to pins 1, 2 and 3 respectively of J4 on the dongle. (Note, that if pins 2 and 3 on J3 are connected, it is possible to force a reset of the MSP430FR2433 from the PC, but the manual reset button will then not be functional. J2 brings out the serial signals, which may be useful if debugging is required for some reason.).

Open UniFlash on your PC. It should automatically recognize your programmer and the attached MSP430 microcontroller. Under “Choose Your Device” select “MSP430FR2433” (not the bootloader version). Select the USB port and click start. Under the Program menu select the forth-mecrisp-msp430fr2433.hex file downloaded from the Mecrisp repository. Under the Settings & Utilities menu tick the box labelled “On connect, erase user code and unlock the device” and make sure that “Erase main and information memory” is selected. Go back to the Program menu and click “Load Image”.

After the successful installation it should now be possible to communicate with the Forth system via the USB port. You can test this with a serial terminal emulator program on the PC, such as CoolTerm (https://freeware.the-meiers.org). The settings are a baudrate of 115200, 8 data bits 8, no parity and 1 stop bit. The USB port has to be selected according to your system. In the *Transmit* menu set the transmit character delay to 3 ms and the transmit line delay to 100 ms. This will allow the microcontroller sufficient time to process the Forth words sent to it.

After the communication has been established pressing the return key should produce an OK on the PC screen. Typing “words CR” should bring up a list of the available Forth words for use.

Now the Forth programming for the SCD41-D-R1 CO_2_ sensor (Forth-Words.txt obtained from the file repository) is installed on the microcontroller. Simply download the file to the microcontroller by making use of the file send facility of CoolTerm. You can check on the success of this step by typing in “words CR” and confirming that the last entry is “read-values”.

The dongle is now ready. It is possible to interact with it using the terminal program, but the Python GUI provides a more convenient user interface.

### 5.4 Setting up the graphical user interface on the PC

Download and install the latest version of Python 3 from https://www.python.org/. The required packages (*matplotlib*, *numpy*, *pyserial*) can then be installed using the package installer *pip,* which is included in the recent versions of Python. Copy the unzipped GUI directory downloaded from the file repository onto your computer. Do not change the directory structure. The GUI is started up by running the Python script GUI.py. It was successfully tested under MS Windows and macOS and should also run under Linux.

## 6 Operation instructions

**Fig. 5:**
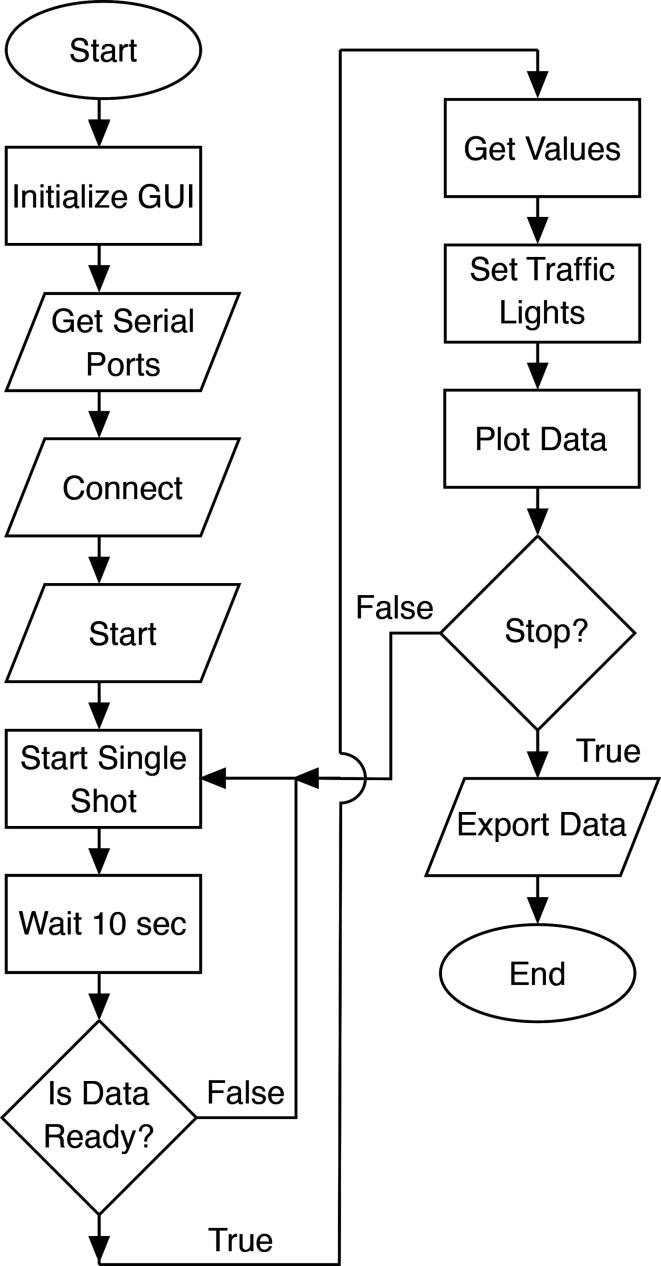
Flow-chart of the main GUI software.

The sequence of the overall operation of the GUI software on the PC is shown in form of a flow-chart in Fig. 5. Before starting up the GUI make sure that the *CO_2_-dongle* is not connected software-wise to your computer anymore via the terminal emulator program (use the *Disconnect* menu item in CoolTerm or close the program). Note, that if the dongle is connected to the computer after starting up the software, the list of COM ports shown on the GUI must be updated by clicking on the refresh button. Choose the COM port used by the dongle, and establish the connection by clicking on the icon with the two plugs. A continuous measurement is started by clicking the green play button. The CO_2_ level (measured every 10 s, rolling average of 5 measurements) is plotted in real time in the chart while the latest measured values for CO_2_, temperature and relative humidity (which are also provided by the CO_2_-sensor itself as auxiliary information) are displayed on the right. Above it, the air quality with respect to the CO_2_ level is indicated by a virtual traffic light: red > 2100 ppm, orange > 1000 ppm, else green. The 1000 ppm threshold was chosen as this corresponds to the general guidelines for upper indoor concentrations [1], but both cut-off levels may be adjusted by the user by making changes to the software. The stop button interrupts the measurements. The small toolbar can be used to navigate in the chart as well as to save the displayed chart as an image. In addition, the CO_2_ data can be exported as a.csv file for further analysis.

## 7 Validation and characterization

As mentioned above, the CO_2_ sensor is designed so that it does not need to be calibrated by the user, but it has a built in self-calibration mechanism which requires the sensor to be exposed to ambient air at least once per week. The accuracy and precision of the measurements are thus determined by the performance of the sensor and not dependent on the user, as it contains its own electronics and the data is given out already in the digital domain. The circuitry and software reported herein therefore has no effect on the quality of the data. The data sheet specifies an accuracy of ±(40 ppm + 5 % of the reading). However, for characterization readings were compared with those of a professional CO_2_-measuring instrument (Extech CO240, www.extechcom). This instrument is specified with an accuracy of ±(75 ppm + 5 % of the reading). A series of readings was obtained by placing both instruments into a box in which an initial high level of CO_2_ was produced by exhaling into it. The box was then closed, but not perfectly sealed, which led to a gradual decline of the CO_2_ concentration inside over a period of about 2 h during which readings were taken. The results are given in the table below.

**Table:** 

Dongle readings (ppm)	CO_2_-Meter readings (ppm)
3533	3112
3115	2734
2229	2164
1841	1642
1504	1343
1113	1024
1033	967
832	800
708	690
599	597
510	525

As can be seen, initially readings above 3000 pmm were obtained. At this high level the two measurements were deviating by about 10 %, with the dongle reading being higher, which is just within the specifications of the two devices. As the CO_2_-level dropped, the readings track each other well, with the 10 % deviation diminishing for the readings below about 1000 ppm.

**Fig. 6:**
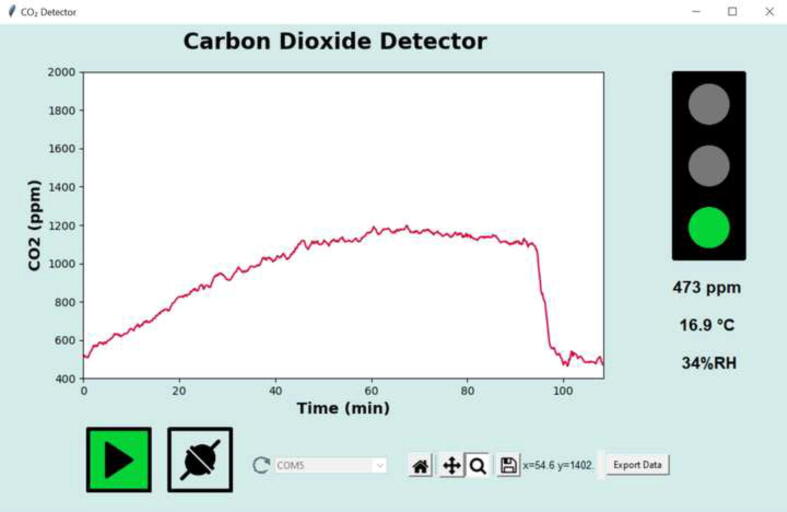
Screen shot of the GUI showing a measurement over a period of just under two hours.

Fig. 6 shows a screen shot from the PC display of a typical CO_2_ measuring session. This was carried out in a closed room with two persons present. At the beginning of the measurement the CO_2_ concentration was close to the ambient air level, but was then continually rising due to the respiration of the people. On opening the window about 90 min after starting the measurement the CO_2_ concentration dropped sharply as the air in the room was being exchanged. Note, that besides the current reading for CO_2_ also the temperature and relative humidity are displayed. These parameters are also determined by the CO_2_ sensor employed. The “traffic light” shows green as the level is below the threshold value to orange of 1000 ppm. In a long term test, the sensor and the software were found to work flawlessly over a period of at least two weeks (the PC must be prevented from hibernating).

Specifications table.

**Table:** 

Hardware name	CO_2_-dongle
Subject area	• General
Hardware type	• Field measurements and sensors
Closest commercial analogue	Desktop CO_2_-monitoring instrument
Open Source License	CERN OHL v.2.0 (https://ohwr.org/project/licences/wikis/home).
Cost of Hardware	appr. € 80
Source File Repository	https://doi.org/10.5281/zenodo.6302824

## Declaration of Competing Interest

The authors declare that they have no known competing financial interests or personal relationships that could have appeared to influence the work reported in this paper.
