# Supplementary figures and images for: CO2-measuring dongle
Source: HardwareX. 2022 Jul 14;12:e00338. doi: 10.1016/j.ohx.2022.e00338 (PMC9301570; doi:10.1016/j.ohx.2022.e00338)

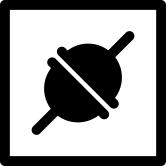

Supplement: Supplementary data 1 [file mmc1.zip › GUI/connected_image.png]

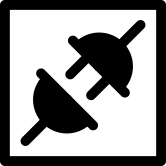

Supplement: Supplementary data 1 [file mmc1.zip › GUI/disconnected_image.png]

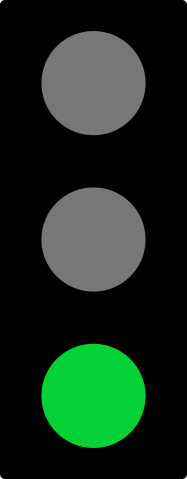

Supplement: Supplementary data 1 [file mmc1.zip › GUI/green_light_image.png]

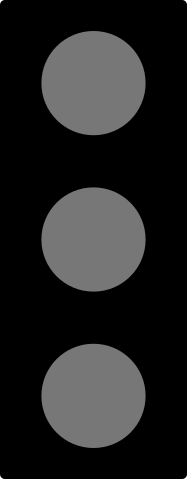

Supplement: Supplementary data 1 [file mmc1.zip › GUI/grey_light_image.png]

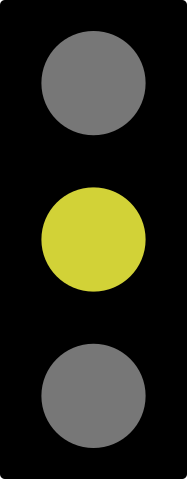

Supplement: Supplementary data 1 [file mmc1.zip › GUI/orange_light_image.png]

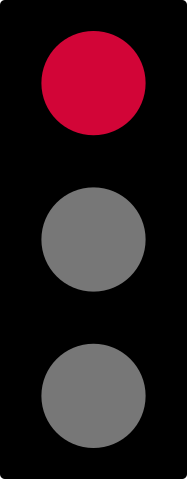

Supplement: Supplementary data 1 [file mmc1.zip › GUI/red_light_image.png]

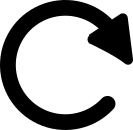

Supplement: Supplementary data 1 [file mmc1.zip › GUI/refresh_image.png]

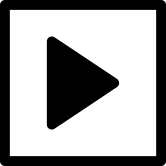

Supplement: Supplementary data 1 [file mmc1.zip › GUI/start_image.png]

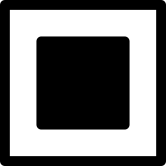

Supplement: Supplementary data 1 [file mmc1.zip › GUI/stop_image.png]
